# Supplementary material for: Aicardi Syndrome Is a Genetically Heterogeneous Disorder
Source: Genes (Basel). 2023 Jul 31;14(8):1565. doi: 10.3390/genes14081565 (PMC10454071; doi:10.3390/genes14081565)
Supplement: Supplementary file 1 [file genes-14-01565-s001.zip › genes-2488016-supplementary/Ha.Aicardi.Supplemental/supplementary Figures.pdf]

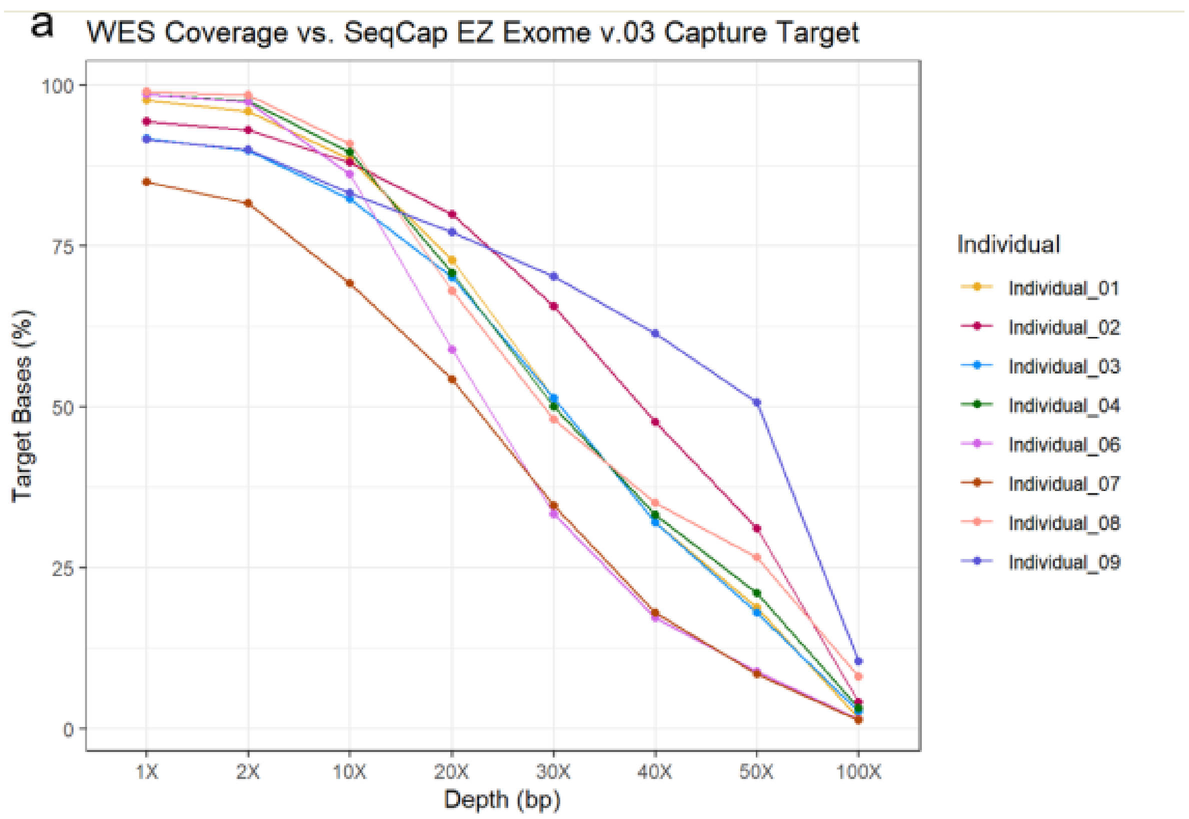

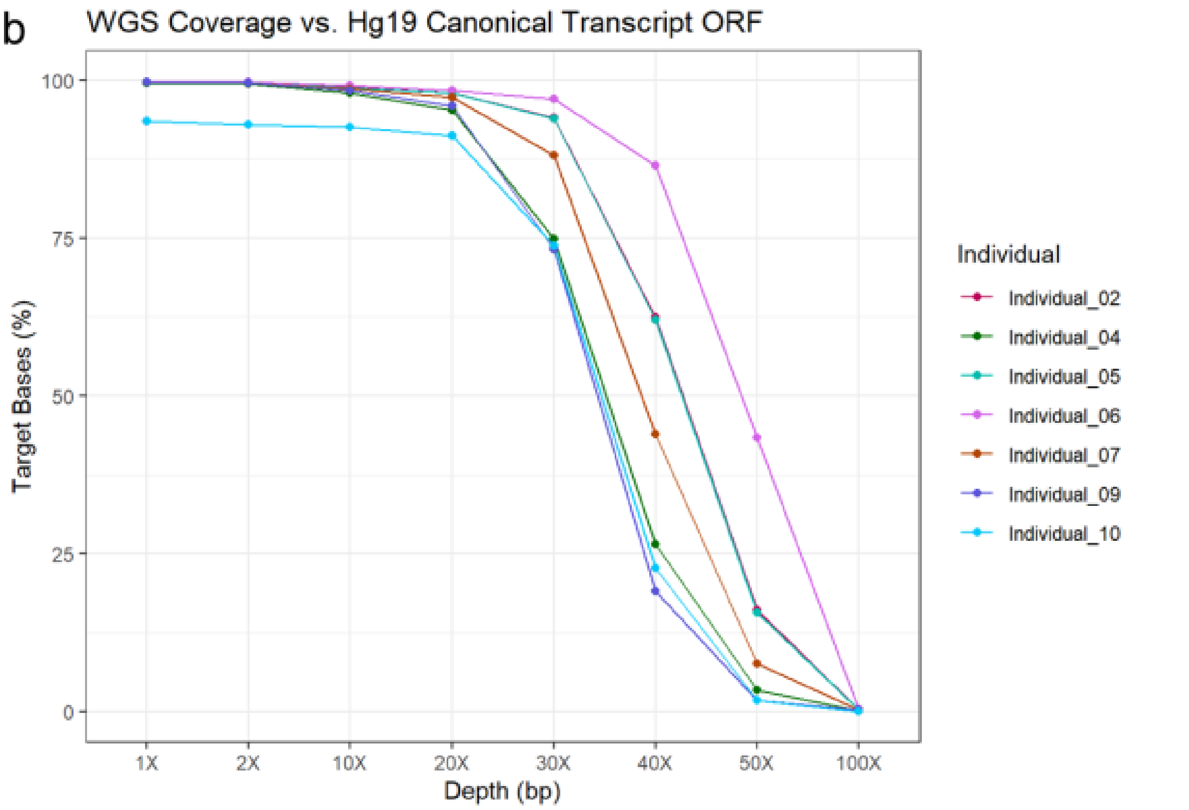

Supplementary Figure S1. Coverage of ES and GS. Supplemental. Sequencing coverage depth compared to the percentage of target bases covered for **a**. SeqCap EZ Exome v3 targets for ES and **b.** hg 19 canonical transcripts open reading frames for GS.

i

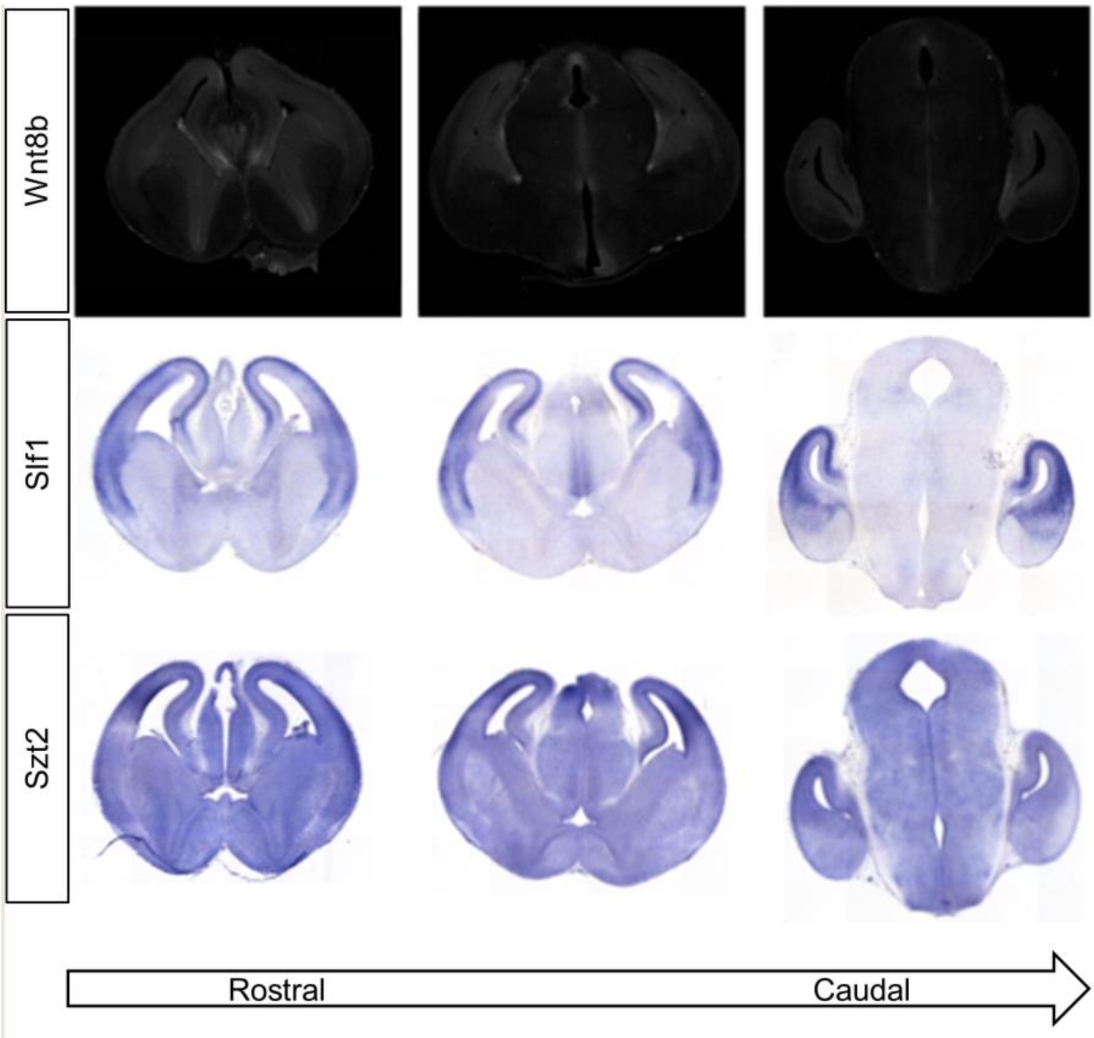

Supplemental Figure S2: Candidate gene expression in ED14.5 mouse brain sections. Coronal 20 µm frozen sections of mouse brain harvested at embryonic day 14. **a.** Top shows expression of Wnt8b measured by indirect immunofluorescent staining. Detection by rabbit anti-WNT8B(Thermo Fisher #PA5-33117) primary antubody. Nuclei in the same section visualised by DAPI staining are shown un the second roe to define morphology of the section. **b.** Detection of S1f1 transcripts by in situ hybridisation using an in vitro transcibed anti-sense riboprobe. **c.** Detection of Szt2 transcripts by in situ hybridisation using an in vitro transcibed anti-sense riboprobe.
